# The Local Treatment of Pain in Gout

**Published:** 1908-07-04

**Authors:** J. Lindsay


### The General Practitioner's Column.

[Contributions to this Column are invited, and if accepted will be paid for.]

### THE LOCAL TREATMENT OF PAIN IN GOUT.

By J. LINDSAY, M.D., Edin.

In addition to the general medicinal treatment of gout, either in its acute or chronic stages, by means of aperients, colchicum, guaiacum, iodide of potassium, and the alkalies potash and lithia, much may be done by local measures to alleviate the often intolerable pain. The most obvious point to attend to is to rest the affected part, and to avoid any pressure over the seat of pain. If the lower limb is affected, then it must be raised, thereby relieving congestion and so diminishing pain. All applications over a joint, the seat of gouty pain, should be warm. In the acute forms especially it is highly inadvisable to employ any radical measures such as local blood-letting, blisters, etc.; by far the more satisfactory results are obtained by applying fomentations over the joints. Simple fomentations are always very grateful to the patient, and are uniformly successful in allaying pain. Other warm applications which should be tried are a solution of acetic acid ʒij., spir. vini. rect. ʒiss., and liq. ammon. acet. ℥iv., made up to a pint with warm water, applied on lint and covered over with oiled silk. Another very useful lotion contains ʒj. each of spir. vini. rect. and sulphuric ether, made up to ℥vj. with warm water and applied as a fomentation. Still another contains morph. hydrochlor. gr.v. and lin. belladon. ℥j., made up to ʒvj. with warm water. These fomentations should be renewed every two or three hours. A preparation which seems to have a particularly comforting influence in gouty pain is the ext. belladonn. virid. made up into the consistence of a paint, with equal portions of glycerine and water. This is applied by soaking lint in the paint and placing over the joint.

Other applications which are found to be of special service in the relief of joint pain, and are employed not only in the treatment of gout, but also of rheumatoid arthritis, are: A pigment consisting of 1 part of salicylate of methyl to 2 parts of olive oil; the unguentum crédé, a somewhat expensive, but highly efficacious, local remedy, containing collargol 3 parts, cera alba 2 parts, and adeps benzoata 15 parts. This, when applied on lint, is a very valuable application in the more chronic forms of joint pain. Cocaine made up with lanolin—gr.iiss. to ℥j.—is useful, more especially in gouty pain affecting the small joints. Of pigments, that in most common use contains equal parts of the tincture and liniment of iodine.

The dull aching pains occurring all over the body, so frequently met with in chronic gout are best treated by rubbing in the lin. terebinth. acet. Sinapisms may also be applied to the larger joints in these conditions. The pain on the under aspect of the heels, which is so frequently present as to be almost pathognomonic of chronic gout, is best treated by painting with hydrochloric acid—a glass brush being used. The skin over this region is so thick that no dilution of the acid is necessary.

As a rule it is recommended that no effort should be made to remove tophi. This may be so in the majority of cases, but in those where the tophi from some reason or another are the seats of pain, or are so situated, as over an interphalangeal joint, as to hinder movement, then it may be justifiable to make an incision and enucleate them. If ordinary care be exercised there need be no fear of sepsis. I have seen quite a number of cases in which this has been done, and in no case was there any untoward result, nor did the tophus in any instance ever return to its former size, even after the lapse of several years. The healing process also is completed sooner than one might perhaps expect in dealing with impaired tissues.

In the joint pains of chronic gout there are few remedies as reliable as blisters; leeches also should be used when other local applications fail, as they are liable to do in the more intractable cases.

Œdema may be localised to one limb—an arm or leg—but is more frequently found in both lower limbs, giving rise to an aching stiffness not complained of in the œdemas of renal, hepatic, or cardiac diseases. Relief here is speedily effected by simple massage in an upward direction and then bandaging the limb upwards towards the trunk. When the œdema tends to persist in the tissues around the ankles, strapping the joints should be resorted to. Wherever gout is settled in the extremities the pain and local condition as a whole will be much benefited by hot-air or vapour baths.

Of the former the electric hot-air baths on the Greville system are the best; than these there is no safer or surer way of administering dry heat. The temperature is under complete control, and can be raised or lowered at will. Special forms of apparatus can be obtained to fit on to any part of the body—both legs, one leg, foot, arm, forearm, shoulder, and so on. So also with vapour baths; these can be applied locally by suiting the apparatus to the needs of the patient; any part of the body, even a single joint, can be exposed to vapour at a temperature varying between 105° to 115° F.

